# Water and Nitrogen Management at the Booting Stage Affects Yield, Grain Quality, Nutrient Uptake, and Use Efficiency of Fragrant Rice Under the Agro-Climatic Conditions of South China

**DOI:** 10.3389/fpls.2022.907231

**Published:** 2022-06-13

**Authors:** Siying Deng, Umair Ashraf, Mohsin Nawaz, Ghulam Abbas, Xiangru Tang, Zhaowen Mo

**Affiliations:** ^1^State key Laboratory for Conservation and Utilization of Subtropical Agro-Bioresources, College of Agriculture, South China Agricultural University, Guangzhou, China; ^2^Scientific Observing and Experimental Station of Crop Cultivation in South China, Ministry of Agriculture, Guangzhou, China; ^3^Division of Science and Technology, Department of Botany, University of Education, Lahore, Pakistan; ^4^College of Agriculture, Hainan University, Haikou, China; ^5^Plant Physiology Section, Agronomic Research Institute, Ayub Agricultural Research Institute, Faisalabad, Pakistan

**Keywords:** biomass, grain yield, nutrient, rice quality, water management

## Abstract

The present study was conducted to assess the effects of water and nitrogen applications at the booting stage on yield, grain quality, and nutrient use efficiencies in fragrant rice in the early (March–July) and late (July–November) seasons of 2013. The experiment was comprised of two fragrant rice cultivars, i.e., Nongxiang 18 and Basmati; three nitrogen levels, i.e., 0 kg N ha^−1^ (N0), 30 kg N ha^−1^ (N1), and 60 kg N ha^−1^ (N2); and three water levels, i.e., 2–4 cm water layer well-watered (W0), water with a soil water potential of −15 ± 5 kPa (W1), and water with a soil water potential of −25 ± 5 kPa (W2), which were randomized in a split-split plot design. Results showed that Basmati produced a higher grain yield than Nongxiang 18 (16.20 and 9.61% in the early and late season, respectively), whereas the W1 exhibited the maximum grain yield and harvest index. The moderate application of nitrogen (N1) at the booting stage resulted in higher grain yield, nevertheless, cultivar, water, and nitrogen revealed different trends for some of the grain quality attributes, i.e., brown rice rate, milled rice rate, head milled rice rate, protein content, and amylose content as well as nutrient uptake and use efficiencies in the double rice production system. Basmati had a higher nitrogen harvest index (NHI; 18.28–20.23%) and P harvest index (PHI; 3.95–12.42%) but lower physiological P use efficiency for biomass (PPUEB; 7.66–23.66%) and physiological K use efficiency for biomass (PKUEB; 2.53–7.10%) than Nongxiang 18 in both seasons. Furthermore, the grain number per panicle, biomass yield, grain P uptake, and the whole plant P uptake were significantly related to the grain yield of fragrant rice. In both seasons, the interaction of water and nitrogen (W × N) had a significant effect on panicle number, grain quality attributes, and N, P uptake of straw, as well as the physiological N, P use efficiency for grain and the physiological N, K use efficiency for biomass. Overall, results suggest that moderate nitrogen and irrigation input at the booting stage could be feasible to improve the productivity and quality of the double rice production system with improved nutrient use efficiency under the agro-climatic conditions of South China.

## Introduction

China contributes about 30% of global rice production wherein fragrant rice is popular due to its pleasant aromatic character and excellent cooking qualities (Bryant and McClung, 2011; Mo et al., 2015; Ashraf and Tang, 2017; Dias et al., 2021). Fragrant rice is traded worldwide at a premium price due to its excellent cooking qualities (Sakthivel et al., 2009), thus increasing the farmers’ interest in enhancing areas under fragrant rice cultivation in China (Li et al., 2022; Lin et al., 2022). Fragrant rice is an ancient, well-known rice type that has secured a leading position due to end-user preference for its aroma and other qualities (Fu et al., 2021; Xie et al., 2021). However, regional climate variabilities as well as management and genetic factors not only affect growth and productivity but also regulate the quality of the fragrant rice (Singh et al., 2005, 2006; Ashraf et al., 2017; Li et al., 2021a,b). Moreover, substantial improvements have also been made to enhance the productivity and quality of fragrant rice by integrating advanced agricultural practices with modern plant breeding approaches (Sautter et al., 2006; Zhang et al., 2021).

Furthermore, efficient nutrient management could also be important to improve the productivity and quality of fragrant rice (Li et al., 2021a,b). Nitrogen (N), which is an important plant nutrient component, ranged from 2.17 to 52.61 mg/g in leaves with 17.55 mg/g dry weight in the north–south transect of eastern China (Ren et al., 2007). Moreover, the nitrogen content of japonica and indica rice was 27 and 25 g/kg dry weight, respectively, which can be used as a critical index of nitrogen deficiency in rice (Wang et al., 2016). Furthermore, the grain quality of rice was found to be strongly linked with nitrogen fertilization (Ishfaq et al., 2020), whereas N application also exhibits a considerable role in the milling quality of rice and aggravates the lusciousness of cooked rice (Champagne et al., 2009). The increase in protein content and decrease in amylose content as a result of N application makes the cooked rice hard, scabrous, and sticky in texture and increased the gel cohesiveness (Gunaratne et al., 2011; Singh et al., 2011), and such characteristics are therefore dependent on N-fertilizer application and the characteristics of cultivars (Hussain et al., 2014).

On the other hand, increased nitrogenous fertilizer application rates did not achieve high utilization efficiency in China, where this was found to be 20–30% lower than the world’s average (Yan et al., 2017). The average N application rate for rice is 180 kg N ha^−1^ year^−1^ in China, but in some parts, it exceeded 450 kg N ha^−1^ (Zhang and Li, 2003; Miao et al., 2011; Zhao et al., 2012; Ju et al., 2015). During the last three decades, the average amount of nitrogen fertilizer applied in South China was 284 kg ha^−1^ (Huang et al., 2020). However, some unreasonable applications of nitrogen fertilizer phenomena limit the optimization of nitrogen fertilizer uptake and utilization efficiency (Ju and Gu, 2014; Zhao et al., 2015), which might be attributed to farmers’ traditional ideas and lack of interest in utilizing advanced agronomic management measures (Yao et al., 2018; Li et al., 2020). Moreover, excessive use of N fertilizers in fragrant rice promotes vegetative growth and reduces the seed set and/or grain formation (Singh et al., 2017). The low nitrogen use efficiency (NUE) in the rice production system has not only decreased the stability of the rice yield but also increased the burden on environmental sustainability (Azusa et al., 2016).

In addition to N, there is no doubt that water management is crucial in the rice production systems of South China. The area using the double-cropping rice system in South China is about 620 × 10^4^ hm^2^ (Ai et al., 2014), whereas the demand for water irrigation is 135 mm for early rice and 265 mm for late rice in the region of South China (Luo et al., 2022). However, excess application of water and low water use efficiencies have always been problems in rice production systems (Hamoud et al., 2019). Various water-saving technologies have recently been developed in rice-based cropping systems to improve water use efficiencies as well as to maintain and/or improve rice productivity (Li, 2006; Sagwal et al., 2022). In this regard, alternate wetting and drying (AWD) is one of the best methods to improve water use efficiency and cost-effective rice production (Bouman and Tuong, 2001; Ashraf et al., 2018). Its application largely depends on the weather conditions and/or soil water contents/potential. Based on Emergy analysis, the double-cropping rice pattern in South China produces less environmental pressure and greater potential for sustainable development (Feng et al., 2019). So, unlike genetic, the agronomical and crop management factors thus largely affect the productivity, quality, and resource use efficiencies of rice-based cropping systems.

Moderate water-nitrogen interaction is beneficial to reducing a series of ecological and environmental problems (He et al., 2016) as well as regulating nitrogen within the rhizosphere and root ecological environment (Wu et al., 2021). Compared with local farmers’ practice (LFP), field-specific nitrogen management and irrigation with alternate wetting and moderate drying not only improved grain yield but also nitrogen and water use efficiency (Xue et al., 2013). Meanwhile, water and nitrogen fertilizer management had altered the magnitude of nitrogen, phosphorus, and potassium uptake in rice; significant synergistic effects of nitrogen, phosphorus, and potassium under the condition of water and nitrogen interaction were even observed (Sun et al., 2011; Yu et al., 2019). Although water and nitrogen management measures are important management for fragrant rice production, there is still a lack of specific information in this regard. Therefore, the present study was conducted to determine the relationships between N levels and water regimes for possible contemporary improvements in the yield, quality, and water and nitrogen use efficiencies as well as to evaluate the relationships between grain yield and nutrient uptake and use efficiencies in the double rice production system in South China.

## Materials and Methods

### Experimental Site, Description, and Design

Field experiments were conducted over two seasons—during the early (March–July) and late (July–November) seasons in 2013—at the Experimental Research Farm, College of Agriculture, South China Agricultural University (SCAU), Guangzhou, China (23°09′N, 113°22′E and 11 m above the sea level). This region has a sub-tropical type of climate with an annual average temperature between 21 and 29°C with 70–80% relative humidity (RH). The experimental farm has been under paddy cultivation for many years. The soil properties of the experimental site and meteorological data are been presented in Tables 1 and 2, respectively.

**Table 1:** Properties of the experimental field soil during both the early and late seasons of the double rice cropping system in South China.

Growing seasons	Organic matter (g kg^−1^)	Total N (g kg^−1^)	Total P (g kg^−1^)	Total K (g kg^−1^)	Available N (mg kg^−1^)	Available P (mg kg^−1^)	Available K (mg kg^−1^)
Early season	23.3	1.1	1.1	24.4	114.3	61.3	127.0
Late season	25.7	1.4	1.0	17.5	85.5	25.1	153.2

**Table 2:** The meteorological data of the experimental site during the early and late seasons of the double rice cropping system in South China.

Month	Mar.	Apr.	May.	Jun.	Jul.	Aug.	Sep.	Oct.	Nov.
Temperature (°C)	19.6	20.9	25.6	28	27.9	27.9	27	23.6	19.5
Humidity (%)	80	86	86	81	82	83	79	66	71
Rainfall (mm)	177.1	268.4	302.7	229.1	273.3	396.6	203.7	5.9	40.3
Sunshine hours (h)	86.8	38.8	73.9	167.3	177.3	162.3	176.1	224.4	150.1

Two popular fragrant rice cultivars, i.e., Nongxiang 18 and Basmati, were grown during both early and late seasons. The N, P_2_O_5_, and K_2_O were applied at 90, 90, and 195 kg ha^−1^ in the form of urea, calcium superphosphate, and potassium chloride, respectively, as basal doses. Additional doses of N were applied at the tillering at 30 kg N ha^−1^. The experimental treatments comprised of three N levels, i.e., 0 kg N ha^−1^ (N0), 30 kg N ha^−1^ (N1), and 60 kg N ha^−1^ (N2), and three levels of water treatments, i.e., 2-4 cm water layer well-watered (W0), water with a soil water potential of −15 ± 5 kPa (W1), and water with a soil water potential of −25 ± 5 kPa (W2) at the booting stage. The water treatments were in accordance with Yang et al. (2007). The treatment period for both seasons was 30 days, i.e., 12 May to 12 June for the early season and 1 September to 1 October 2013 for the late season. Apart from the treatment period, all plots were flooded 3 days after transplanting with a water depth of 2–4 cm for 7 days before maturity. The pest and weed control were implemented according to the guidelines for fragrant rice cultivation in South China (Tang et al., 2014).

The treatments were randomized in a split-split plot design with cultivars in the main plot, water levels in sub-plots, and nitrogen levels in sub-sub plots in triplicate. The plot size was 3 m × 5 m (375 hills plot^−1^). Seedlings from early rice (21 days old) and late rice (17 days old) from wet bed nurseries were transplanted at a rate of 2 seedlings per hill at a 20 cm × 20 cm (2.5 × 10^5^ hills ha^−1^) planting distance on 31 March and 2 August and harvested on 12 July and 1 November for the early and late seasons, respectively. The nursery of the late season develops earlier and grows faster than the early season due to the natural climatic conditions of the region (Li et al., 2016).

#### Sampling and Data Collection

At maturity, 25 hills from each plot were harvested in triplicate, threshed manually, and three 100 g samples were oven-dried at 105°C to constant weight to calculate the water content and then converted into 14% grain moisture content. Panicles were threshed manually from five random hills to count the total and filled grain percentage. A rice blower and husk separator were used to separate the filled and empty hulls, and the seed setting rate was calculated as the filled grains/total number of grains × 100. Five samples of 1,000 grains were taken randomly from the filled seed lot and weighed to get the 1,000-grain weight (g). The panicle numbers were counted from 1 m^2^ to measure the number of panicles per m^2^, and then five hills were separated into leaves, stems, and grains for dry matter determination and nutrient (N, P, and K) accumulation and distribution analyses. The plant parts were dried to a constant weight in an oven at 80°C. For nutrient measurement in plant tissues, oven-dried samples were ground into powder and digested and analyzed for N, P, and K by the method described by Lu (1999). The digestion was then used to determine the total N content by the Kjeldahl method with a 2300 Kjeltec Analyzer Unit (Foss Tecator AB, Swedish). The P and K contents were determined by using the UV-VIS Spectrophotometer UV-2550 (SHIMADZU, Japan) and the Atomic Absorption Spectrophotometer AA-6300C (SHIMADZU, Japan) method, respectively.

The harvest index was calculated as (grain yield/aboveground dry biomass) × 100%. The physiological N, P, and K use efficiency for grain (biomass) was defined as the weight of grain (biomass) divided by total N, P, and K uptake (Jiang et al., 2004). The N, P, and K harvest index was defined as the total N, P, and K uptake in grain divided by total N, P, and K in the whole plant (Albrizio et al., 2010).

The grains (500 g) from the harvested seed lot (stored at room temperature in a well-aerated storage room for at least 3 months) were taken for quality analyses. The brown rice rate was tested with a rice huller (Jiangsu, China) whereas the milled rice and head milled rice rates were estimated with a Jingmi testing rice grader (Zhejiang, China) and calculated as follows:

Brown rice rate = brown rice weight/rice sample weight × 100%
Milled rice rate = milled rice weight/rice sample weight × 100%.
Head milled rice rate = milled rice weight/rice sample weight × 100%.

The amylose and protein contents of grains were measured by using an Infratec 1241 grain analyzer (FOSS-TECATOR).

#### Data Analysis

The data analyses and correlation coefficients were estimated with Statistix version 8 (Analytical, and Tallahassee, Florida, United States) whilst the differences amongst treatments were separated by the least significant difference (LSD) test at a 0.05 level of significance.

## Results

### Dry Biomass, Yield, and Yield Components

For water treatments, the W1 had the highest grain yield in the early season (748.24 g m^−2^), followed by the late season (621.14 g m^−2^). Compared with W0, the grain yield was increased by 30.57 and 21.83% under W1 and W2 treatments during the early season whilst it was decreased by 0.18 and 5.11% under the same water treatments during the late season, respectively. However, the reduction in grain yield was found non-significant for W1 in the late season rice. Compared to N0, N1, and N2 significantly increased grain yield by 7.72 and 6.28%, respectively, in the late season rice. The improvement of grain yield was also observed for N1 and N2 in the early season but remained non-significant. Moreover, Basmati showed a significantly higher grain yield than Nongxiang 18 (16.20 and 9.61% in the early and late seasons, respectively). Moreover, Basmati had a lower panicle number (2.13 and 4.84%) and low harvest index (2.13 and 4.84%) than Nongxiang 18 but had a higher grain number per panicle (25.15 and 12.03%), higher straw weight (20.37 and 9.05%), and a higher biomass yield (18.18 and 15.89%) in the early and late season, respectively. Regarding cultivars, the seed setting rate and 1,000 grain weight showed an opposite trend for both seasons. Reduced water levels resulted in higher panicle numbers, grain numbers per panicle, and biomass yield in the early season than well-water treatments, however, an opposite trend was observed for the late season. The straw weight was found the highest under W2 treatment in the early season (914.58 g m^−2^) whilst lower than control in the late season. The N1 and N2 resulted in an increased panicle number, straw weight, and biomass yield but decreased grain number per panicle and seed setting rate. The 1,000 grain weight increased with increasing nitrogen levels, but the opposite trend was investigated for harvest index in the early season. The N1 treatment had the highest 1,000 grain weight (28.03 g) and harvest index (43.39%) in the late season (Tables 3 and 4). In addition, water and nitrogen (W × N) were found statistically significant (*P*<0.05) regarding panicle number, grain number per panicle, seed setting rate, and 1,000 grain weight as well as straw weight and biomass yield in the early season and only for panicle numbers in the late season.

**Table 3:** Grain yield and yield components as affected by water, nitrogen, and cultivars in the early and late seasons of double rice cropping system in South China.

Treatment		Grain yield (g m^−2^)	Panicle number per m^2^	Grain number per panicle	Seed setting rate (%)	1,000 grain weight (g)
*Early season*						
Cultivar	Nongxiang 18	622.54 b	199.44 a	148.22 b	77.474 a	27.587 a
	Basmati 385	723.75 a	195.19 b	185.50 a	77.228 a	26.914 b
Water	W0	573.06 b	190.83 c	157.64 b	70.086 b	27.342 a
	W1	748.24 a	195.56 b	180.42 a	79.565 a	27.131 b
	W2	698.15 a	205.56 a	162.53 b	79.403 a	27.274 ab
Nitrogen	N0	666.07 a	184.17 c	171.72 a	78.834 a	27.014 b
	N1	668.37 a	201.39 b	166.74 b	76.318 a	27.343 a
	N2	685.01 a	206.39 a	162.12 c	76.901 a	27.394 a
ANOVA	Water (W)	45.01^**^	39.95^**^	34.06^**^	11.92^*^	4.29 ns
	Nitrogen (N)	0.49 ns	69.99^***^	3.52 ns	2.40 ns	21.61^***^
	W × N	2.00 ns	7.84^**^	10.34^***^	5.79^**^	37.19^***^
	Cultivar (C)	31.54^***^	11.02^**^	120.46^***^	0.09 ns	253.17^***^
	W × C	8.53^**^	56.02^***^	2.57 ns	17.30^***^	6.67^**^
	N × C	1.22 ns	11.08^***^	3.10 ns	1.49 ns	54.88^***^
	W × N × C	1.07 ns	0.96 ns	1.05 ns	2.37 ns	22.92^***^
*Late season*						
Cultivar	Nongxiang 18	583.24 b	237.15 a	124.28 b	79.172 b	27.121 b
	Basmati 385	639.30 a	225.67 b	139.22 a	85.869 a	28.138 a
Water	W0	622.24 a	233.17 a	139.15 a	80.288 b	27.542 a
	W1	621.14 a	232.33 a	132.17 b	85.541 a	27.748 a
	W2	590.43 b	228.72 a	123.94 c	81.732 b	27.598 a
Nitrogen	N0	584.00 b	214.83 c	134.33 a	83.312 a	27.502 b
	N1	629.11 a	232.61 b	131.09 a	82.928 a	28.034 a
	N2	620.70 a	246.78 a	129.84 a	81.321 a	27.353 b
ANOVA	Water (W)	7.00^*^	1.10 ns	41.48^**^	9.46^*^	2.16 ns
	Nitrogen (N)	15.54^***^	79.37^***^	2.36 ns	2.00 ns	12.46^**^
	W × N	2.55 ns	9.09^**^	0.85 ns	2.23 ns	3.18 ns
	Cultivar (C)	94.85^***^	29.57^***^	59.98^***^	62.24^***^	258.61^***^
	W × C	26.37^***^	7.67^**^	1.46	4.26^*^	4.33^*^
	N × C	28.45^***^	19.25^***^	5.96^*^	1.67 ns	1.49 ns
	W × N × C	8.96^***^	1.23 ns	3.46^*^	1.33 ns	24.40^***^

Different low case letters indicate the significant difference among treatments at 0.05 level. N0: 0 kg N ha^−1^, N1: 30 kg N ha^−1^, N2: 60 kg N ha^−1^. W0: well-watered, W1: soil water potential was −15 ± 5 kPa, W2: soil water potential was −25 ± 5 kPa. *Significant at p < 0.05, **Significant at p < 0.01, ***Significant at p < 0.001, and ns: non-significant.

**Table 4:** Straw weight, biomass yield, and harvest index as affected by water, nitrogen, and cultivar in the early and late seasons of double rice cropping system in South China.

Treatment		Strew weight(g/m^2^)	Biomass yield (g/m^2^)	Harvest index (%)
*Early season*				
Cultivar	Nongxiang 18	785.09 b	1405.7 b	44.67 a
	Basmati 385	945.01 a	1661.3 a	43.68 a
Water	W0	847.32 b	1455.1 c	39.73 b
	W1	833.25 b	1494.0 b	50.52 a
	W2	914.58 a	1651.5 a	42.28 b
Nitrogen	N0	827.15 b	1511.7 b	44.70 a
	N1	854.55 b	1512.1 b	44.19 a
	N2	913.45 a	1576.8 a	43.64 a
ANOVA	Water (W)	16.20^*^	158.24^***^	28.62^**^
	Nitrogen (N)	9.04^**^	17.89^***^	0.26 ns
	W × N	3.52^*^	7.22^**^	1.92 ns
	Cultivar (C)	253.09^***^	528.49^***^	0.61 ns
	W × C	12.47^***^	68.21^***^	0.01 ns
	N × C	21.21^***^	22.94^***^	4.40^*^
	W × N × C	30.17^***^	52.69^***^	4.31^*^
*Late season*				
Cultivar	Nongxiang 18	871.23 b	1337.9 b	43.86 a
	Basmati 385	950.12 a	1550.5 a	41.52 b
Water	W0	964.83 a	1552.6 a	40.51 c
	W1	910.71 ab	1455.1 b	42.78 b
	W2	856.48 b	1324.9 c	44.77 a
Nitrogen	N0	854.62 b	1375.6 b	42.84 a
	N1	929.33 a	1453.6 a	43.39 a
	N2	948.08 a	1503.4 a	42.83 a
ANOVA	Water (W)	13.15^*^	51.06^**^	21.28^**^
	Nitrogen (N)	9.34^**^	8.64^**^	1.07 ns
	W × N	1.61 ns	1.06 ns	0.76 ns
	Cultivar (C)	8.89^**^	54.14^***^	5.76^*^
	W × C	2.83 ns	6.43^**^	2.59 ns
	N × C	0.81 ns	1.52 ns	4.63^*^
	W × N × C	2.98^*^	2.03 ns	0.86 ns

Different lower case letters indicate the significant difference among treatments at 0.05 level. N0: 0 kg N ha^−1^, N1: 30 kg N ha^−1^, N2: 60 kg N ha^−1^. W0: well-watered, W1: soil water potential was −15 ± 5 kPa, W2: soil water potential was −25 ± 5 kPa. *Significant at p < 0.05, **Significant at p < 0.01, ***Significant at p < 0.001, and ns: non-significant.

### Correlation Between Yield and Yield Components and Biomass

**Figure 1:**
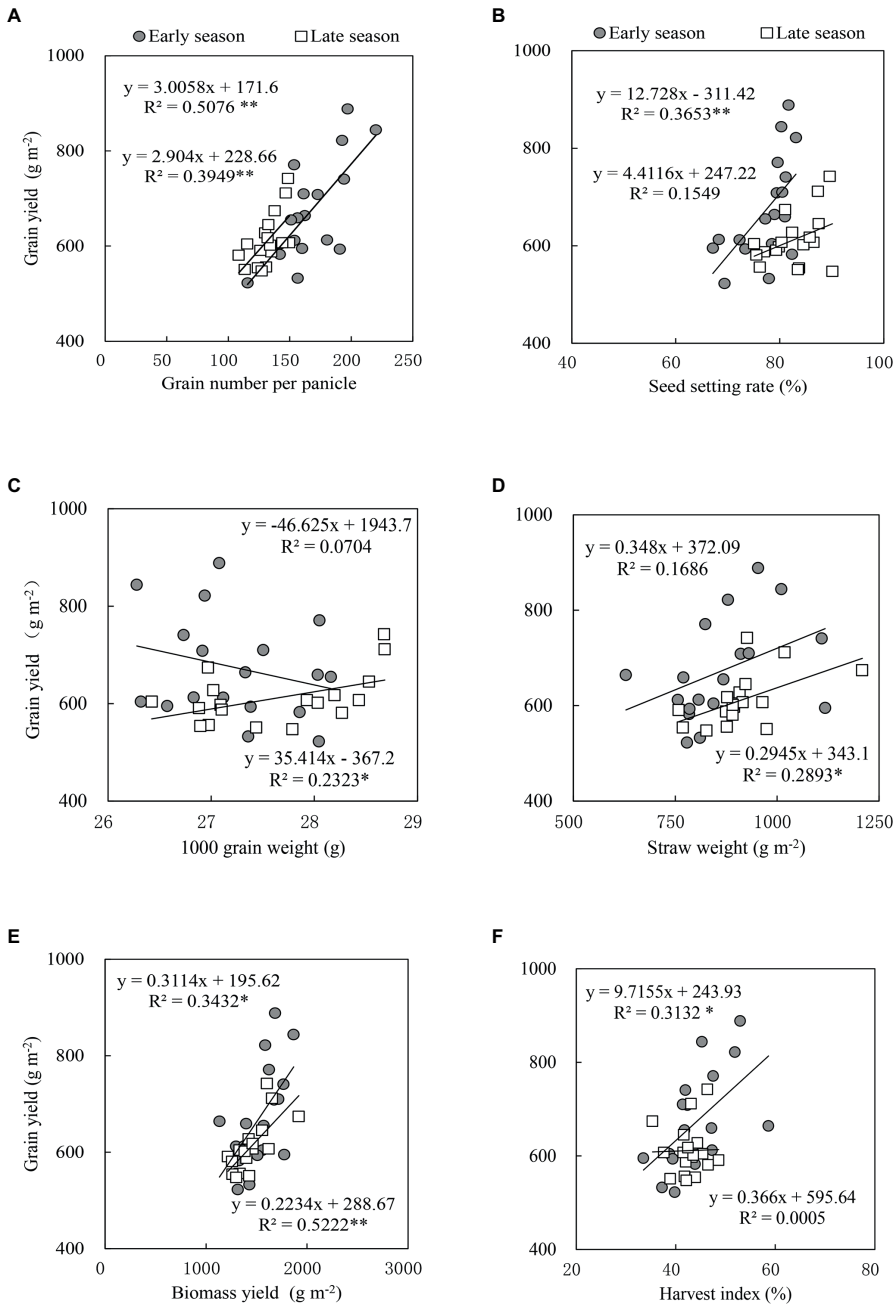
Relationships between grain yield with **(A)** the number of grains per panicle, **(B)** seed setting rate %, **(C)** 1,000-grain weight, **(D)** straw weight, **(E)** biomass yield, and **(F)** harvest index of the early and late season of double rice cropping system in South China. ^**^Significant at *p* < 0.05; ^*^Significant at *p* < 0.01. A smaller *R*^2^ value means greater variability.

Grain number per panicle and biomass yield were significantly and positively correlated with grain yield in both the early and late season (Figures 1A,E), however, the grain yield was significantly related to seed setting rate and harvest index only for the early season (Figures 1B,F). Further, significant and positive associations of grain yield with 1,000 grain weight and straw weight were also recorded for the late season (Figures 1C,D).

### Grain Quality Attributes

Basmati was found to be superior to Nongxiang 18 in the late season regarding the brown rice rate, whereas reduced irrigations increased the brown rice rate for the early season while results were found otherwise for the late season. The N2 had the highest brown rice rate, i.e., 80.22 and 83.14% in the early and the late season, respectively. The Nongxiang 18 had a substantially higher milled rice rate than Basmati for the early season, while it was lower for the late season. The milled rice rate significantly increased with the decrease in water for the early season, while an opposite trend was found for the late season. Likewise, the milled rice rate significantly decreased with the increase in nitrogen for the early season, while an opposite trend was found for the late season. Furthermore, W1 and W2 had significantly higher head milled rice rates than W0 in the early season whereas the N0 and N1 resulted in higher head milled rice rates than N2. However, a substantial increase in head milled rice rate was recorded with the increase in nitrogen for the late season. The Nongxiang 18 had a significantly lower protein content than Basmati in the early season. The protein content significantly increased under WI and W2 water treatments than W0 for the early season, while W2 showed significantly higher protein than W1 for the late season. The highest protein content was recorded in N1, i.e., 7.08 and 9.01% in the early and the seasons, respectively, compared to N0. The trend of amylose contents under different N treatments was recorded as N0 ˃ N2 ˃ N1 (Table 5). In addition, water and nitrogen interaction (W × N) was found statistically significant (*p* < 0.05) regarding brown rice rate (%), milled rice rate (%), head milled rice rate (%), the protein content (%) and amylose content (%) in the early season. However, water and nitrogen interaction (W × N) was found statistically significant (*p* < 0.05) regarding all quality traits except for brown rice rate in late season rice (Table 5).

**Table 5:** Grain quality attributes as affected by water, nitrogen, and cultivar in early and late seasons of double rice cropping system in South China.

Treatment		Brown rice rate (%)	Milled rice rate (%)	Head milled rice rate (%)	Protein content (%)	Amylose content (%)
*Early season*						
Cultivar	Nongxiang 18	80.00 a	67.90 a	49.77 a	6.79 b	23.77 a
	Basmati 385	80.06 a	66.97 b	39.69 b	6.94 a	23.67 a
Water	W0	79.39 b	66.16 c	41.87 b	6.60 c	23.81 a
	W1	80.37 a	67.83 b	45.90 a	6.68 b	23.61 a
	W2	80.32 a	68.31 a	46.41 a	7.31 a	23.74 a
Nitrogen	N0	79.99 b	67.61 a	46.89 a	6.78 b	24.42 a
	N1	79.87 b	67.56 a	47.19 a	7.07 a	22.45 b
	N2	80.22 a	67.13 b	40.11 b	6.73 b	24.28 a
ANOVA	Water (W)	226.22^***^	399.51^***^	361.00^***^	497.70^***^	0.64 ns
	Nitrogen (N)	15.65^***^	11.55^**^	518.15^***^	56.36^***^	62.20^***^
	W × N	5.33^*^	10.10^***^	64.62^***^	223.97^***^	48.16^***^
	Cultivar (C)	0.70 ns	110.66^***^	1626.78^***^	42.11^***^	0.88 ns
	W × C	6.83^**^	0.53 ns	9.36^**^	1168.92^***^	2.24 ns
	N × C	5.80^*^	2.57 ns	11.86^***^	169.29^***^	98.69^***^
	W × N × C	15.15^***^	32.83^***^	22.00^***^	611.51^***^	254.14^***^
*Late season*						
Cultivar	Nongxiang 18	82.22 b	68.04 b	61.11 b	9.02 a	18.87 a
	Basmati 385	83.05 a	69.85 a	64.37 a	8.67 b	18.89 a
Water	W0	82.79 a	69.21 a	62.73 a	8.83 ab	18.75 a
	W1	82.71 a	68.94 b	62.99 a	8.78 b	18.81 a
	W2	82.41 b	68.68 c	62.50 a	8.93 a	19.07 a
Nitrogen	N0	82.14 c	68.62 b	61.83 c	8.91 b	18.94 a
	N1	82.63 b	68.78 b	62.68 b	9.01 A	18.71 a
	N2	83.13 a	69.43 a	63.71 a	8.63 c	18.93 a
ANOVA	Water (W)	8.75^*^	18.96^**^	0.70 ns	7.00^*^	0.79 ns
	Nitrogen (N)	18.06^***^	28.94^***^	18.71^***^	212.80^***^	0.38 ns
	W × N	3.23 ns	10.27^***^	5.78^**^	109.43^***^	17.56^***^
	Cultivar (C)	50.44^***^	366.15^***^	119.28^***^	307.20^***^	0.01 ns
	W × C	0.41 ns	0.56 ns	4.70^*^	140.40^***^	10.12^**^
	N × C	3.71^*^	9.20^**^	2.23 ns	20.80^***^	17.68^***^
	W × N × C	3.10^*^	1.81 ns	4.11^*^	147.70^***^	21.96^***^

Different low case letters indicate the significant difference among treatment at 0.05 level. N0: 0 kg N ha^−1^, N1: 30 kg N ha^−1^, N2: 60 kg N ha^−1^. W0: well-watered, W1: soil water potential was − 15 ± 5 kPa, W2: soil water potential was − 25 ± 5 kPa. *Significant at p < 0.05, **Significant at p < 0.01, ***Significant at p < 0.001, and ns: non-significant.

### N, P, and K Uptake

Basmati showed higher grain N uptake (14.06–60.76%), grain P uptake (32.83–72.85%), straw P uptake (25.63–38.89%), whole plant P uptake (28.61–53.10%), grain K uptake (5.60–25.79%), straw K uptake (20.03–31.18%), and whole plant K uptake (20.58–28.40%) than Nongxiang 18 in both seasons. However, a lower straw N uptake and whole plant N uptake were detected for Basmati compared to Nongxiang 18 in the late season (Table 6).

**Table 6:** The N, P, and K uptake in grain, straw, and the whole plant as affected by water, nitrogen, and cultivar in the early and late seasons double rice cropping systems in South China.

Treatment		GNU (g m^−2^)	SNU (g m^−2^)	WPNU (g m^−2^)	GPU (g m^−2^)	SPU (g m^−2^)	WPPU (g m^−2^)	GKU (g m^−2^)	SKU (g m^−2^)	WPKU (g m^−2^)
*Early season*										
Cultivar	Nongxiang 18	3.63 b	6.39 b	10.02 b	1.66 b	2.34 b	4.01 b	2.73 a	22.42 b	25.16 b
	Basmati 385	5.83 a	8.00 a	13.84 a	2.21 a	2.94 a	5.16 a	2.89 a	29.41 a	32.30 a
Water	W0	3.83 c	6.62 b	10.45 c	1.73 b	2.44 c	4.18 c	2.61 b	25.59 ab	28.20 b
	W1	4.64 b	7.01 b	11.66 b	1.99 a	2.67 b	4.67 b	2.74 b	24.44 b	27.19 b
	W2	5.72 a	7.96 a	13.68 a	2.09 a	2.82 a	4.92 a	3.08 a	27.71 a	30.79 a
Nitrogen	N0	5.06 a	7.16 b	12.23 a	1.92 a	2.38 c	4.30 b	2.75 ab	22.65 b	25.40 c
	N1	4.51 b	6.86 b	11.38 b	1.86 a	2.51 b	4.37 b	2.68 b	26.80 a	29.49 b
	N2	4.61 b	7.57 a	12.18 a	2.04 a	3.05 a	5.09 a	3.00 a	28.29 a	31.29a
ANOVA	Water (W)	282.92^***^	13.23^*^	108.78^***^	14.82^*^	70.98^****^	63.24^***^	11.05^*^	8.00^*^	10.73^*^
	Nitrogen (N)	7.05^**^	9.53^**^	20.77^***^	2.36 ns	72.84^***^	68.67^***^	3.22 ns	25.93^***^	29.18^***^
	W × N	1.98 ns	51.08^***^	59.85^***^	0.22 ns	11.34^***^	8.70^**^	2.62 ns	8.19^**^	8.50^**^
	Cultivar (C)	275.13^***^	116.25^***^	375.80^***^	68.50^***^	112.77^***^	230.10^***^	2.38 ns	76.73^***^	88.33^***^
	W × C	25.71^***^	44.17^***^	71.78^***^	8.10^**^	8.22^**^	8.84^**^	2.80 ns	2.83 ns	3.91^*^
	N × C	11.01^***^	1.21 ns	4.95^*^	8.96^**^	18.24^***^	12.53^***^	7.54^**^	12.85^***^	14.51^***^
	W × N × C	15.55^***^	24.76^***^	1.34 ns	3.91^*^	12.19^***^	18.48^****^	1.55	30.51^***^	34. 26^***^
*Late season*										
Cultivar	Nongxiang 18	3.59 b	6.60 a	10.19 a	1.40 b	1.90 b	3.31 b	1.87 a	17.49 b	19.36 b
	Basmati 385	4.09 a	5.51 b	9.61 a	2.42 a	2.64 a	5.07 a	2.35 a	20.99 a	23.35 a
Water	W0	4.14 a	6.94 a	11.09 a	1.94 ab	2.54 a	4.48 a	2.53 a	21.12 a	23.65 a
	W1	3.92 a	6.20 b	10.12 b	2.10 a	2.21 b	4.31 a	2.07 b	18.31 b	20.36 b
	W2	3.46 b	5.02 c	8.49 c	1.69 b	2.07 b	3.77 b	1.74 b	18.28 b	20.05 b
Nitrogen	N0	3.89 ab	5.29 c	9.19 b	1.84 a	1.99 c	3.84 b	1.83 a	18.58 a	20.42 a
	N1	3.61 b	6.87 a	10.49 a	1.95 a	2.33 b	4.29 a	2.19 a	19.11 a	21.30 a
	N2	4.04 a	6.01 b	10.03 a	1.94 a	2.49 a	4.43 a	2.31 a	20.02 a	22.34 a
ANOVA	Water (W)	8.92^*^	46.96^**^	89.50^***^	8.78^*^	11.66^*^	55.44^**^	18.24^**^	10.76^*^	21.78^**^
	Nitrogen (N)	3.45 ns	19.75^***^	10.82^**^	0.71 ns	27.03^***^	12.77^**^	2.34 ns	1.58 ns	2.33 ns
	W × N	5.61^**^	5.59^**^	6.18^**^	2.40 ns	15.67^***^	1.87 ns	0.37 ns	4.23^*^	3.84^*^
	Cultivar (C)	8.85^**^	11.54^**^	2.76 ns	131.71^***^	123.30^***^	249.42^***^	4.40 ns	40.89^***^	48.32^***^
	W × C	8.32^**^	5.96^*^	2.03 ns	5.17^*^	7.64^**^	11.97^***^	1.85 ns	40.18^***^	42.72^***^
	N × C	1.29 ns	9.90^**^	11.53^***^	8.32^**^	10.07^**^	16.97^***^	0.69 ns	19.88^***^	18.27^***^
	W × N × C	14.35^***^	2.15 ns	4.78^**^	1.00 ns	6.69^**^	0.95 ns	0.99 ns	46.56^***^	40.39^***^

GNU, SNU, WPNU, GPU, SPU, WPPU, GKU, SKU, and WPKU represent Grain N uptake, Straw N uptake, Whole plant N uptake, Grain P uptake, Straw P uptake, Whole plant P uptake, Grain K uptake, Straw K uptake, and Whole plant K uptake, respectively. Different low case letters indicate the significant difference among treatments at 0.05 level. N0: 0 kg N ha^−1^, N1: 30 kg N ha^−1^, N2: 60 kg N ha^−1^. W0: well-watered, W1: soil water potential was −15 ± 5 kPa, W2: soil water potential was −25 ± 5 kPa. *Significant at p < 0.05, **Significant at p < 0.01, ***Significant at p < 0.001, ****Significant at p< 0.0001, and ns: non-significant.

The grain N uptake, straw N uptake, whole plant N uptake, straw P uptake, whole plant P uptake, and grain K uptake increased with the decrease in water during the early season, while an opposite trend was seen during the late season. The grain P uptake increased with the decrease in water during the early season, but the straw K uptake and the whole plant K uptake decreased with the decrease in water during the late season. In addition, the highest grain P uptake (2.10 g m^−2^) was observed in W1 during the late season, while the highest straw K uptake (27.71 g m^−2^) and the whole plant K uptake (30.798 g m^−2^) were recorded in W2 during the early season (Table 6).

The straw and whole plant P and K uptake increased with the increase in nitrogen application for both seasons. The highest grain K uptake was observed in N2, i.e., 3.00 and 2.32 g m^−2^ for the early and late seasons, respectively. In addition, water and nitrogen interaction (W × N) was found to be statistically significant (*p* < 0.05) regarding SNU, WPNU, SPU, WPPU, SKU, and WPKU in the early season rice and regarding GNU, SNU, WPNU, SPU, SKU, and WPKU in the late season rice (Table 6).

### N, P, and K Use Efficiency

Basmati had a higher NHI (18.28–20.23%) and P harvest index (PHI; 3.95–12.42%) but lower PPUEB (7.66–23.66%) and physiological K use efficiency for biomass (PKUEB; 2.53–7.10%) than Nongxiang 18 in both seasons. Opposite trends were observed regarding PNUEG, PNUEB, PPUEG, and PKUEG, and the K harvest index (KHI) was recorded for both rice cultivars. The NHI and PPUEG increased with the decrease in irrigations for both seasons, whilst the PNUEB decreased with decreased water application rate for the early season while the opposite was seen for the late season. The PPUEG and PHI were decreased with the increase in nitrogen application (Table 7). In addition, water interaction with nitrogen (W × N) was found to be statistically significant (*p* < 0.05) regarding PNUEG, PNUEB, NHI, PPUEG, PPUEB, and PKUEB in early season rice. However, water interaction with nitrogen (W × N) was found to be statistically significant (*p* < 0.05) regarding PNUEG, PNUEB, NHI, PPUEG, PHI, and PKUEB in late season rice.

**Table 7:** Physiological N, P and K use efficiency for grain and biomass and N, P, and K harvest index as affected by water, nitrogen, and cultivar in the early and late season of double rice cropping system in South China.

Treatment		PNUEG (g g^−1^)	PNUEB (g g^−1^)	NHI (%)	PPUEG (g g^−1^)	PPUEB (g g^−1^)	PHI (%)	PKUEG (g g^−1^)	PKUEB (g g^−1^)	KHI (%)
*Early season*										
Cultivar	Nongxiang 18	63.909 a	142.60 a	35.896 b	142.22 b	353.05 a	41.48 a	25.72 a	57.57 a	11.21 a
	Basmati 385	52.978 b	123.23 b	42.457 a	157.60 a	325.99 b	43.12 a	23.55 b	53.49 b	9.36 b
Water	W0	55.241 b	140.21 a	36.591 b	140.83 b	352.52 a	41.83 a	21.78 c	54.18 a	9.78 a
	W1	67.654 a	134.54 b	38.748 ab	144.69 b	323.46 b	42.12 a	28.56 a	56.60 a	10.54 a
	W2	52.435 b	124.00 c	42.191 a	164.21 a	342.58 a	42.95 a	23.57 b	55.80 a	10.53 a
Nitrogen	N0	57.354 a	127.52 b	40.549 a	160.57 a	358.24 a	44.00 a	27.05 a	60.49 a	11.02 a
	N1	59.643 a	135.50 a	39.508 ab	152.50 a	347.58 b	42.46 ab	23.28 b	52.94 b	9.50 b
	N2	58.333 a	135.73 a	37.472 b	136.66 b	312.73 c	40.44 b	23.58 b	53.16 b	10.33 ab
ANOVA	Water (W)	56.88^**^	48.28^**^	9.70^*^	15.88^*^	13.67^*^	0.65 ns	213.94^***^	1.03 ns	2.48 ns
	Nitrogen (N)	0.67 ns	25.68^***^	3.09 ns	10.64^**^	58.42^**^8	2.76 ns	7.88^**^	18.42^***^	3.66 ns
	W × N	6.42^**^	105.67^***^	10.23^***^	6.60^**^	13.58^***^	1.22 ns	1.78 ns	5.96^**^	3.18 ns
	Cultivar (C)	34.75^***^	105.19^***^	67.58^***^	9.69^**^	25.29^***^	2.37 ns	5.45^*^	8.97^**^	12.47^**^
	W × C	10.99^***^	49.69^***^	0.46 ns	2.31 ns	12.54^***^	6.83^**^	2.71 ns	7.12^**^	0.20 ns
	N × C	2.30 ns	1.86 ns	3.88^*^	7.46^**^	2.68 ns	9.57^**^	10.43^***^	8.26^**^	3.22 ns
	W × N × C	1.77 ns	21.09^***^	37.28^***^	6.47^**^	2.62 ns	0.22 ns	14.68^****^	22.94^***^	6.72^**^
*Late season*										
Cultivar	Nongxiang 18	59.587 b	135.03 b	35.817 b	177.85 a	406.79 a	42.553 b	23.233 b	71.871 a	10.06 a
	Basmati 385	68.919 a	166.37 a	43.061 a	128.57 b	310.53 b	47.838 a	31.407 a	70.050 a	10.54 a
Water	W0	57.489 b	142.95 b	38.048 a	146.57 b	360.72 a	42.771 a	29.052 b	70.856 a	11.29 a
	W1	63.181 b	148.20 b	38.948 a	151.17 b	354.21 a	48.286 a	30.694 ab	73.554 a	10.47 a
	W2	72.089 a	160.95 a	41.322 a	161.88 a	361.04 a	44.530 a	31.124 a	68.471 a	9.14 a
Nitrogen	N0	65.923 a	154.87 a	42.714 a	155.28 a	362.63 a	47.731 a	30.226 a	70.088 a	9.27 a
	N1	63.408 a	145.53 b	35.214 b	152.31 a	353.50 a	44.572 ab	30.383 a	70.624 a	10.89 a
	N2	63.428 a	151.69 ab	40.389 a	152.02 a	359.84 a	43.284 b	30.351 a	72.168 a	10.73 a
ANOVA	Water (W)	21.49^**^	18.24^**^	2.60	9.92^*^	0.67 ns	4.01	4.21 ns	3.06 ns	1.81 ns
	Nitrogen (N)	0.79 ns	3.20 ns	16.67^***^	0.28 ns	0.37 ns	4.66^*^	0.01 ns	0.80 ns	1.81 ns
	W × N	3.92^*^	7.53^**^	6.63^**^	3.63^*^	2.93 ns	8.31^**^	1.99 ns	3.49^*^	0.58 ns
	Cultivar (C)	13.44^**^	65.08^***^	20.00^***^	133.23^***^	266.04^***^	14.91^**^	7.15^*^	0.96 ns	0.21 ns
	W × C	3.26 ns	7.39^**^	11.27^***^	0.28 ns	8.75^**^	1.48	25.60^***^	18.56^***^	0.57 ns
	N × C	11.91^***^	19.56^***^	3.96^*^	7.56^**^	26.06^***^	2.10	23.65^***^	9.80^**^	1.61 ns
	W × N × C	4.37^*^	14.03^***^	7.91^***^	2.02 ns	7.10^**^	4.13^*^	27.24^***^	34.91^***^	5.27^**^

PNUEG, PNUEB, NHI, PPUEG, PPUEB, PHI, PKUEG, PKUEB, and KHI represent physiological N use efficiency for grain, physiological N use efficiency for biomass, N harvest index, physiological P use efficiency for grain, physiological P use efficiency for biomass, P harvest index, physiological K use efficiency for grain, physiological K use efficiency for biomass, and K harvest index, respectively. Different low case letters indicate the significant difference among treatments at 0.05 level. N0: 0 kg N ha^−1^, N1: 30 kg N ha^−1^, N2: 60 kg N ha^−1^. W0: well-watered, W1: soil water potential was −15 ± 5 kPa, W2: soil water potential was −25 ± 5 kPa. *Significant at p < 0.05, **Significant at p < 0.01, ***Significant at p < 0.001, ****Significant at p < 0.0001, and ns: non-significant.

### Correlation Analyses Between Yield NPK Uptake and Use Efficiency

**Figure 2:**
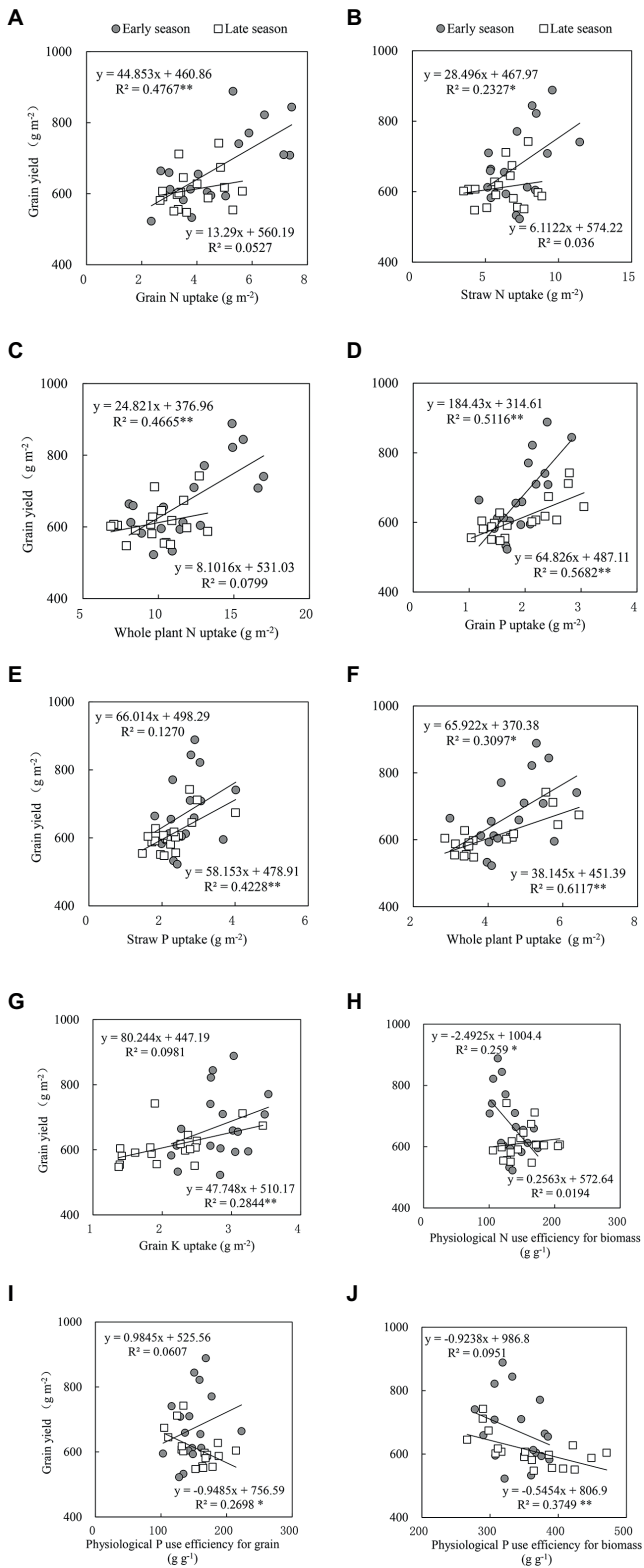
Relationship between grain yield and **(A)** grain N uptake, **(B)** straw N uptake, **(C)** whole plant N uptake, **(D)** grain P uptake, **(E)** straw P uptake, **(F)** whole plant P uptake, **(G)** grain K uptake, **(H)** physiological N use efficiency for biomass, **(I)** physiological P use efficiency for grain, and **(J)** physiological P use efficiency for the biomass of both the early and late season double rice cropping systems in South China. ^**^Significant at *p* < 0.05; ^*^Significant at *p* < 0.01. A smaller *R*^2^ value means greater variability.

Significant relationships were observed between grain yield and grain P uptake and the whole plant P uptake for both seasons. For the early season, the grain yield was significantly related to grain N uptake, straw N uptake, whole plant N uptake, and PNUEB. During the late season, grain yield was positively associated with straw P uptake, grain K uptake, PPUEG, and PPUEB for the late season (Figure 2).

## Discussion

This study investigated the effects of different levels of water and nitrogen application at the booting stage on yield, grain quality, and nutrient use efficiencies in fragrant rice during the early and late seasons of the double rice cropping system in South China. Nutrient management is an important aspect of aromatic rice production whereas the application of organic/inorganic manure/fertilizers at an appropriate dose is necessary to get higher yields with improved grain quality characters (Paul et al., 2021). In this study, the grain yield for late season rice was remarkably affected by changing the nitrogen (N) and water levels at the booting stage, and Basmati showed a significantly higher grain yield than Nongxiang 18, while the water and nitrogen interaction (W × N) had no significant effect on grain yield during both seasons (Table 3). However, Zhou et al. (2006) reported the positive coupling effect of water and fertilizer on rice yield as well as improved heading rate and biomass yield of rice (Wu et al., 2020). Moreover, both rice cultivars remained substantially different regarding panicles number, grain number per panicle, grain weight, straw weight, and biomass yield in both seasons (Tables 3 and 4), whereas Basmati produced more grain per panicle (25.15% in the early season and 12.03% in late season), higher straw weight (20.37% in early season and 9.05% in the late season), and a higher biomass yield (increased by 18.18 and 15.89% during the early and late seasons, respectively) than Nongxiang 18 (Table 3). Our findings corroborate with those of Laenoi et al. (2017) and Fageria et al. (2010) who found seasonal variations of rice cultivars regarding head rice yield in different rice cultivars.

For grain yield, Mo et al. (2017), Ali et al. (2012), and Ning et al. (2009) reported that urea application increased rice grain yield by 5–18% in the upland rice paddy soils. In the present study, nitrogen has affected the panicles number, grain weight, straw weight, and biomass yield with obvious effects during both seasons (Tables 3 and 4). Additional application of nitrogen increased panicle number, straw weight, and biomass yield but decreased grain number per panicle and seed setting rates. Li et al. (2016) reported a 0–12.9% increase in rice yield with different nitrogen application rates. Additionally, Nakano and Morita (2008) also reported that the increased amount of nitrogen enhanced the dry matter yield by up to 10%. The difference in dry matter production depends on the effect of nitrogen on leaf production and individual leaf dry weight (Cechin and Fumis, 2004). More grain and biomass yield might be explained by the higher capability of the rice cultivar to utilize more nitrogen through a better growth pattern and more dry matter. It is confirmed that an increase in aboveground-biomass production through the nitrogen application during the reproductive stage is the primary factor in increasing grain number in flooded rice (Chen et al., 2012). The integrative crop management with judicious use of the N fertilizer not only increased grain yield, NUE, but also enhanced agronomic performance with an improved tillering ability (Zhang et al., 2005). Undoubtedly, a large number of rice varieties is reported to produce non-productive tillers, which limit the rice grain yield (Peng et al., 1999). Fageria (2007) reported that improved crop production through an increase in plant density generally results in a dense crop stand, which is more susceptible to lodging and damage from insects and diseases. On the contrary, low plant densities could lead to low grain yield, which is a common problem under the local farmer’s practice in current rice production in China (Chen et al., 2014; Ju et al., 2015). In our study, the plant density is the same for both cultivars but the difference in the performance owing to efficient utilization of the resources.

Furthermore, a positive correlation between grain yield, 1,000 grain weight, and straw weight was observed (Figures 1C,D). However, there was a negative correlation between grain yield and 1,000 grain weight of early rice with a smaller *R*^2^ value (Figure 1C). The difference in growth and yield formation of rice might be due to the variations in external temperature and/or weather conditions. For instance, higher temperature at night decreased yield (90%) by affecting the spikelet sterility (61%), grain length (2%), width (2%), and decreased yield due to increased spike sterility (Prasad et al., 2006; Mohammed and Tarpley, 2010). Xiong et al. (2016) reported that the rice area of South China is high and rainy, which might make early rice precocious in the late grouting stage. Lower dry matter yield is associated with a higher temperature at the heading stage in the early season than in the late season (Kong et al., 2017). Furthermore, a higher brown rice rate was found in Basmati than Nongxiang 18 (Table 5). Laenoi et al. (2017) studied four different rice cultivars and reported that the “Pathum Thani 1” rice cultivar produced a substantially higher rice yield than all other cultivars and found a negative correlation between the number of grains, tillers, and panicles. Increased sink capacity in the rice plant, i.e., panicles and spikelet per unit area, is the presumption of higher crop yield (Xu et al., 2005;Zhang et al., 2013).

Moreover, reduced amylose contents (the early season 22.46% and the late season 18.76%) was observed in N1, and the interaction of water-nitrogen (W × N) also significantly affected protein content and amylose content in both seasons (Table 6). In general, the hardness of the grain is associated with the higher amylose contents in grains and more glutinous (Singh et al., 2003; Koutroubas et al., 2004). Rice quality attributes such as the protein content in a head rice increased considerably with the decrease in irrigation water. Guo et al. (2020) reported that the ground cover rice production system (GCRPS) reduces irrigation water but increases yield with a slight reduction in grain quality. Simultaneously, head rice percentage and grain protein content were significantly enhanced by N application (Ning et al., 2009). Furthermore, Wopereis-Pura et al. (2002) revealed that different timings of N application also affected the head rice percentage whereas N application at booting and heading stages led to an increase in head rice percentage and grain amylose content.

The improvement in grain yield and yield components with an additional application of N fertilizer at the booting stage might be associated with efficient nutrient uptake. The nitrogen application effect on straw N uptake, the whole plant N uptake, straw P uptake, and the whole plant P uptake was observed notably in both seasons (Table 6). Wopereis-Pura et al. (2002) and Fan et al. (2005) reported the higher application rates resulted in an increase in the N-uptake and whole plant N content. In addition, the N, P, and K use efficiency were remained variable in both experimental seasons under additional N application whilst the significant effects of nitrogen were observed on PNUEB, PPUEG, PPUEB, PKUEG, and PKUEB for the early season rice only (Table 7), where the grain yield showed negative relations with PNUEB and PPUEB of early rice (Figures 2H,J). Plant growth and nutrient use efficiency (NUE) are perceived to be controlled by genetic and physiological behavior and are modified by altering environmental conditions (Xie et al., 2019). The N had a substantial impact on the plant growth and development, followed by P and K, but the balance of the N, P, and K content in rice is frequently disturbed by the external environment (Ma et al., 2022). Pan et al. (2009) reported significant interactions between water and nitrogen on total N uptake and NUE in a 2-year field experiment, but grain yield in the first year was higher than in the subsequent year possibly due to conducive environmental conditions. Moreover, grain yield, water use efficiency, and NUE in rice are greatly determined by irrigation regimes and their interaction with nitrogen rates (Wang et al., 2016; Ku et al., 2017; Yan et al., 2022) whereas the fertilizer use efficiency is greatly influenced by mineral nutrient absorption rate, mobilization, and utilization in plant cultivars (Li et al., 2014).

## Conclusion

Overall, W1 and N2 treatments resulted in a higher rice grain yield. Regarding cultivars, Basmati showed higher grain N uptake, grain P uptake, straw P uptake, whole plant P uptake, grain K uptake, straw K uptake, and whole plant K uptake than Nongxiang 18. However, lower straw N uptake and the whole plant N uptake were detected for Basmati than Nongxiang 18. Meanwhile, Basmati had the higher NHI and PHI but lower PPUEB and PKUEB than Nongxiang 18 in both seasons. In summary, intermittent irrigation with an additional N dose at the booting stage improved the yield and quality attributes as well as NPK uptake and use efficiencies in fragrant rice. This study contains basic information regarding the water and nitrogen regulations at the booting stage and their effect on yield, quality, and nutrient use efficiencies; however, further studies are still needed before any recommendations are made to the farmers.

## Data Availability Statement

The original contributions presented in the study are included in the article/supplementary material, further inquiries can be directed to the corresponding authors.

## Author Contributions

ZM designed the experiments. SD and ZM performed the traits investigation. SD, UA, and ZM analyzed the data and wrote the manuscript. ZM, UA, MN, GA, and XT revised and edited the manuscript. All authors contributed to the article and approved the submitted version.

## Funding

This study was financially supported by the National Natural Science Foundation of China (31271646 and 31601244).

## Conflict of Interest

The authors declare that the research was conducted in the absence of any commercial or financial relationships that could be construed as a potential conflict of interest.

## Publisher’s Note

All claims expressed in this article are solely those of the authors and do not necessarily represent those of their affiliated organizations, or those of the publisher, the editors and the reviewers. Any product that may be evaluated in this article, or claim that may be made by its manufacturer, is not guaranteed or endorsed by the publisher.
